# Latent infection of low-virulence anaerobic bacteria in degenerated lumbar intervertebral discs

**DOI:** 10.1186/s12891-018-2373-3

**Published:** 2018-12-20

**Authors:** Guoqing Tang, Zhuo Wang, Ji Chen, Zhengshi Zhang, Hongbin Qian, Yong Chen

**Affiliations:** Orthopedic Center, Kunshan Hospital of Traditional Chinese Medicine, Kunshan, Suzhou, 215000 China

**Keywords:** Low-virulence anaerobic bacteria, Modic changes, *Propionibacterium acnes*, Coagulase-negative *staphylococci*, Disc herniation

## Abstract

**Background:**

The existence of latent low-virulence anaerobic bacteria in degenerated intervertebral discs (IVDs) remains controversial. In this study, the prevalence of low-virulence anaerobic bacteria in degenerated IVDs was examined, and the correlation between bacterial infection and clinical symptoms was analysed.

**Methods:**

Eighty patients with disc herniation who underwent discectomy were included in this study. Under a stringent protocol to ensure sterile conditions, 80 disc samples were intraoperatively retrieved and subjected to microbiological culture. Meanwhile, tissue samples from the surrounding muscle and ligaments were harvested and cultured as contamination markers. The severity of IVD degeneration and the prevalence of Modic changes (MCs) were assessed according to preoperative MRI analysis.

**Results:**

Of the 80 cultured discs, 54 were sterile, and 26 showed the presence of bacteria: *Propionibacterium acnes* (21 cases) and coagulase-negative *staphylococci* (5 cases). MRI revealed that the presence of bacteria was significantly associated with MCs (*P*<0.001). However, there was no significant association between bacterial infection and the severity of IVD degeneration (*P* = 0.162).

**Conclusions:**

Our findings further validated the presence of low-virulence anaerobic bacteria in degenerated IVDs, and *P. acnes* was the most frequent bacterium. In addition, the latent infection of bacteria in IVDs was associated with Modic changes. Therefore, low-virulence anaerobic bacteria may play a crucial role in the pathophysiology of MCs and lumbar disc herniation.

## Background

Disc degeneration is a multifaceted chronic process that can lead to herniation, radiculopathy, myelopathy, spinal stenosis, and degenerative spondylolisthesis and that alters intervertebral disc structure and function, potentially resulting in acute or chronic pain [1]. However, there is a poor correlation between degenerate disc imaging findings and back pain. While radicular symptoms are often related to nerve root compression by a prolapsed disc, it is not uncommon to observe a discrepancy between the degree of disc prolapse and pain severity [2]. This incongruity between clinical symptoms and imaging findings is likely related to disc inflammation and mechanical injury. Over the last decade, emerging evidence suggests that low-grade infection is another possible factor [3, 4].

Stirling et al. performed microbiological culturing of intervertebral disc material from 36 patients who underwent micro-discectomy for severe sciatica and found that 53% were positive for *Propionibacterium acnes* [3]. Furthermore, serological testing of 140 patients with sciatica revealed that 31% had increased titres of serum immunoglobulin G to the lipid S antigen. Their findings suggested that microbial infection could be responsible for sciatica in disc herniation. Additionally, a recent RCT demonstrated that antibiotic treatment was effective for the treatment of chronic low back pain, implying that disc herniation could be secondary to infection [5].

Approximately 70% of studies report that nonspecific low back pain is positively associated with Modic changes (MCs), i.e., bone oedema in vertebrae, with odds ratios ranging from 2.0 to 19.9 [4, 6]. Moreover, Albert et al. observed new MC development within 1–2 years after discectomy in 80% of patients with anaerobic bacteria detected in their discs, compared to only 44% of patients with negative cultures [4]. Urquhart et al. performed a systematic review of 11 published studies on this topic and found moderate evidence indicating that low-virulence bacteria play a role in disc herniation with low back pain and that bacterial presence is related to MCs associated with disc herniation [7].

Nevertheless, there are still many challenges regarding the topic of low anaerobic bacterial infection in intervertebral discs. For example, there is still not enough clinical research throughout the world to replicate the results and verify the conclusion. Additionally, the lack of reliable contamination control during disc harvest or culture in some studies renders the results less accurate. Additionally, the correlation among bacterial infection, Modic changes and low back pain needs to be investigated with better clinical data. Therefore, the aim of the present study was to analyse the possible presence of bacteria in intervertebral disc biopsies of patients who underwent surgery for lumbar disc herniation and to examine the association between bacterial presence and MCs. Verification of such a link could result in a conceptual paradigm shift and usher in a new era of lumbar disc problem management.

## Methods

### Patients

Eighty consecutive patients from January 2017 to August 2017 who underwent discectomy due to a single level of lumbar disc herniation were included in this study. All of the patients had severe sciatica with or without low back pain. The study was approved by the Institutional Ethics Board, and written informed consent was authorized by all patients. A total of 80 IVDs were collected for bacterial culture, of which 10 were from levels L3–L4, 41 were from L4–L5, and 29 were from L5–S1. The patients received instrumented posterior lumbar fusion after discectomy if they had spinal instability or spondylolisthesis. The patients who had more severe lower back pain than leg pain also underwent lumbar fusion None of the patients had clinical or biological signs of infection before the operation. No patients were given antibiotics within one month prior to the surgery.

### Preoperative imaging

For all patients, preoperative anteroposterior and lateral lumbar spine radiographs were obtained in the standing position. Magnetic resonance imaging (MRI) was performed for 68 patients. The Modic classification system was applied to evaluate signal intensity changes in the adjacent vertebral body based on a previous protocol [8]. The severity of disc degeneration was evaluated according to the Pfirrmann system [9].

### Intraoperative samples

During general anaesthesia induction, all patients received prophylactic antibiotic treatment with a single dose of cefazolin of 2 g following the instructions of the Institutional Ethics Board. Strict aseptic techniques were used for intraoperative disc removal. In brief, the skin of the operation field was disinfected with povidone iodine 3 times, and then the antimicrobial incise drape (3 M Ioban 2 Antimicrobial Incise Drape, 3 M Health Care, St. Paul, MN, USA) was used to cover the surgical field. Subsequently, the wound was irrigated twice using sterilized water before the discectomy of intervertebral discs. Then, the specimen was handled only using a fresh instrument to minimize contamination and was quickly transferred into a sterile pot and immediately covered with the lid. Finally, tissue samples from the surrounding muscles and ligaments were obtained at the same time to serve as the contamination marker.

### Microbiological culture

All of the tissue samples were sent to the Laboratory of Bacteriology in our hospital. Under a class II laminar flow safety cabinet, each sample was dissected into 5 segments with a sterilised scalpel. Three of the 5 segments were plated on three types of culture media plates, and 2 of them were in enriched broth. For plate culture, the dilacerated segments were first spread across the surface of the plates, and then the tissue was embedded into the plate. Briefly, the segments were embedded in MacConkey agar plates that were aerobically incubated at 37 °C (results read at 7 and 14 days), chocolate agar plates incubated at 37 °C in 5% CO_2_ (results read at 7 and 14 days), and horse blood agar plates anaerobically incubated at 37 °C (results read at 7 and 14 days), respectively.

For broth enrichment, one segment was cultured in cooked meat medium broth and incubated at 37 °C. After 48 h of incubation, one broth subculture was plated onto horse blood agar plates and anaerobically incubated at 37 °C or onto chocolate agar plates and incubated at 37 °C in 5% CO_2_, and the results were read 7 days later. A second segment was incubated in cooked meat medium broth for 7 days and then plated onto horse blood agar plates and anaerobically incubated at 37 °C or plated onto chocolate agar plates and incubated at 37 °C in 5% CO_2_, and the results were read 7 days later (14 days from surgery). When the samples had bacterial growth, they were identified with 16Sr DNA PCR with universe primers, and the products of PCR were analysed and compared to known genes.

### Statistical analysis

For continuous variables, the data were expressed as the mean ± SD. A two-sided Student’s t-test was used to analyse differences between the two groups. Categorical variables were compared using Fisher’s exact test and the Chi-square test. *P < 0.05* was considered significantly different.

## Results

A total of 80 patients with 80 disc samples were enrolled in this study. The cohort included 35 males and 45 females, with a mean age of 51 ± 14.9 years. Among the patients, 17.6% had diabetes, and 36.8% were smokers. No patients received preoperative transforaminal, epidural, or facet joint injections before the surgery. No patients had a history of spinal or abdominal surgery. Of the patients, 12 received surgery at the level of L3/4, 40 underwent surgery at L4/5, and 28 underwent surgery at L5/S1.

Microbiological disc cultures revealed that 26 disc samples (26/80, 32.5%) were positive for bacterial growth. However, 3 cases had bacterial growth both in discs and surrounding ligaments or muscles at the same time, and the remaining 23 samples (23/80, 28.7%) only had bacterial growth in the discs. Considering that the 3 discs may have been contaminated during surgery, 23 cases were considered absolute bacteria- positive.

As depicted in Table 1, *P. acnes* was detected in 21 samples (21/80, 26.25%, one case had bacterium growth at both the muscle and ligament), and coagulase-negative *Staphylococcus* (CNS) was detected in 5 samples (5/80, 6.25; two cases had bacteria growth at both the muscle and ligament). Statistical analysis revealed that patients with bacteria- positive in IVDs were significantly younger than bacteria-negative patients (*P* = 0.03, Table 2). There were no significant differences between positive and negative patients in smoking, diabetes and levels of discs (Table 2).

**Table 1:** Relationships between bacteria and severity of disc degeneration

	Disc degeneration		total	*p*
	Positive culture	Negative culture		
Pfirrmann IV	3	15	18	0.162
Pfirrmann V	20	39	59	
Total	23*	54	77	

*Three cases of suspicious contamination were excluded

**Table 2:** Patient and clinical characteristics in relation to bacterial positivity

Parameters	*n* = 77*	Positive culture *n* = 23*	Negative culture *n* = 54	*P*
Age	50.9 ± 12.1	44.7 ± 11.5	53.5 ± 11.5	0.003
Male	35	13	22	0.203
Female	42	10	32	
Diabetes	12	3	9	0.979
Smoker	26	6	20	0.352
VAS for back	4.2 ± 1.8	4.0 ± 1.7	4.2 ± 1.8	0.616
VAS for leg	5.1 ± 1.6	5.1 ± 1.5	5.1 ± 1.7	0.989
Level of disc culture				
L3/4	10	4	6	0.250
L4/5	40	14	26	
L5/S1	27	5	22	

*Three cases of suspicious contamination were excluded

MRI revealed that 25 discs (25/80, 31.2%) had MCs (including Modic change I and Modic change II) at adjacent vertebrae. Of these, 15 cases (15/80, 18.7%) were bacteria-positive, while the remaining 10 cases (10/80, 12.5%) were bacteria-negative. Table 3 shows that there was a significant association between the presence of bacteria and MCs (*P* < 0.01). However, there was no statistical association between bacterial infection and the severity of disc degeneration (*P* = 0.162, Table 1).

**Table 3:** Relationships between bacterial infection and MCs

	Culture in discs		total	*p*
	Positive culture	Negative culture		
MCs (type I/ II)	15	10	25	<0.001
No MCs	8	44	52	
Total	23*	54	77	

*Three cases of suspicious contamination were excluded

## Discussion

In recent years, multiple studies have reported low-virulent anaerobic bacteria in disc material, suggesting that such infection may contribute to degenerate spine conditions, including sciatica, neck pain, and back pain. In this study, 32.5% of patients had low-virulence bacteria in the IVDs, which was similar to previous reports of approximately 8%~ 53% [10]. In addition, the surrounding muscles and ligaments served as contamination markers, so the isolated bacteria could be attributed to original growth inside the IVDs rather than possible contamination. Therefore, we concluded that there was latent infection of low-virulence bacteria in IVDs with a prevalence of 28.75% after the exclusion of three suspicious cases.

In addition, the bacteria we isolated and identified were also similar to those in previous reports. In our study, *P. acnes* accounted for 26.25%, and CNS accounted for 6.25%. Stirling et al. first reported that the prevalence of *P. acnes* and CNS was 44.4% (16/36) and 5.5% (2/36) in degenerated IVDs, respectively [3]. Subsequently, several papers also reported the existence of low-virulence anaerobic bacteria in IVDs, especially *P. acnes*, with the prevalence ranging from 45 to 84%. More importantly, the survival, reproduction and pathogenicity of the bacterium was further validated in animal studies according to previous reports [11, 12]. Hence, low-virulence anaerobic bacteria, especially *P. acnes*, have been considered a new pathogenic factor for disc disease by increasing numbers of scholars.

Despite this evidence, the hypothesis that latent infection of anaerobic low-virulence bacteria resided in IVDs remains controversial. Several studies have suggested that bacteria detected in disc cultures may originate from the patient’s skin, the air, and laminar flow, especially that *P. acnes* or CNS are normal flora residing on the human skin [13, 14]. For example, McLorinan et al. demonstrated that *P. acnes* would contaminate the incision during the operation, indicating that the bacteria was detected in 29.1% of skin samples, 21.5% of tissue samples, and 16.5% of washings [14]. However, it is difficult to attribute all the isolated bacteria to contamination because several papers have demonstrated the existence of bacteria within the IVDs, and the phylogroup patterns of bacteria were different from those of skin [10].

Reports by Stirling and others suggest that low-grade infection may play a role in inflammation and herniation [3, 15–17]. In our study, positive cultures were significantly associated with Modic changes in the adjacent vertebrae, further supporting the theory that MCs in the vertebrae adjacent to a previously herniated disc may be related to oedema surrounding an infected disc. Animal studies also demonstrated that inoculation of anaerobic low-virulence bacteria into IVDs would cause degenerative discogenic disease, such as Modic changes or disc degeneration, which is different from the pyogenic discitis caused by *Staphylococcus aureus* (*S. aureus*) [11, 12]. Therefore, low-virulence anaerobic bacteria have been considered one of the reasons for Modic changes.

Unfortunately, there were still several limitations in this study. First, all patients had to receive preoperative prophylactic antibiotics according to the protocol of our Institutional Ethics Board, which may have decreased the rate of positive culture. Additionally, we acknowledge that the use of antibiotics might also have reduced the number of positive cultures obtained from the ligament and muscle material, which was interpreted in this study as a control for contamination. Additionally, the polymerase chain reaction was not performed directly on tissue to detect the bacterial DNA. Although laboratory culture is the gold standard for the diagnosis of bacterial pathogens, polymerase chain reaction has advantages and disadvantages. Finally, more samples should have been included in the study to increase the accuracy of the conclusions.

## Conclusion

In summary, the results of our present study confirmed previous findings of low-grade infection in patients undergoing surgery for a first time disc herniation. Such infections might play important roles in the pathophysiology of MCs and may change the therapeutic strategy in the future.

## Abbreviations

CNS: coagulase-negative *Staphylococcus*
IVDs: intervertebral discs
MCs: Modic changes
*P. acnes*: *Propionibacterium acnes*
